# Vitamin Enhanced Waters and Polyphenol Rich Beverages Analyzed for Antioxidant Capacity and Antioxidants/Calorie

**DOI:** 10.3390/nu2121290

**Published:** 2010-12-15

**Authors:** Patrick E. Donnelly, Thomas M. Churilla, Michael G. Coco, Joe A. Vinson

**Affiliations:** Loyola Hall of Science, Department of Chemistry, Scranton, Pennsylvania, 18510, USA; Email: churillat2@gmail.com (T.M.C.); cocom2@scranton.edu (M.G.C.J.); vinson@scranton.edu (J.A.V.)

**Keywords:** polyphenols, antioxidant capacity, flavonoids, Folin-Ciocalteu

## Abstract

The purpose of this study was to analyze polyphenol rich beverages (vitamin enhanced waters (VEWs), fruit juices and berry juices) to determine free polyphenol concentrations and free polyphenols per Calorie based on a serving size. The Folin–Ciocalteu reagent was used in a colorimetric assay based on a catechin standard. Fruit and berry juices contained, on average, more than eight-times the concentration of free polyphenols when compared to VEWs. When Calories per serving were taken into consideration, fruit and berry juices contained more than twice the free polyphenols per Calorie.

## 1. Introduction

Polyphenols have become a recent interest to health conscious individuals. As a result, beverages containing high concentrations of polyphenols are becoming increasingly popular. This is due to the health benefits that accompany polyphenol consumption, derived from their antioxidant properties [1]. Polyphenols have been found to inhibit reactive oxygen species formation and protect against coronary heart disease [2,3]. Polyphenols have also been reported to potentially inhibit the development of certain types of cancer, such as breast cancer, and slow tumor growth [4,5,6,7,8]. The fact that these polyphenols can help prevent chronic disease has prompted beverage manufacturers to produce drinks that boast antioxidant content and consumers may look to these beverages for consumption of antioxidants and nutrients. These include vitamin enhanced waters (VEWs) and a variety of fruit and berry juices.

Currently, there is a large variety of VEWs on the market and some products advertise high concentrations of antioxidants and therefore high antioxidant capacity. There are also the four berry juices that are thought to be rich in polyphenols: These are noni, goji, mangosteen and acai. Other widely available polyphenol rich beverages are fruit and berry juices, *i.e.*, blueberry, mango, pineapple, *etc.* Some of these juices have been analyzed for their antioxidant content [9]. Consumption of the whole fruits and berries also provide dietary polyphenols. Many fruits and berries have been analyzed for their polyphenol content [10,11]. 

In this study, the concentrations of free polyphenols in VEWs, both regular and two low-calorie varieties, and various fruit and berry juices were determined. The free polyphenols per Calorie were also compared and ranked accordingly among all beverages studied. The study was conducted colorimetrically using the Folin-Ciocalteu reagent. The flavonoid catechin was used as a standard. The study included 25 VEWs of differing brands and 20 fruit and berry juices. The fruit and berry juices were either a single juice or a juice blend, e.g., cranberry-grape. 

## 2. Experimental Section

### 2.1. Sample Preparation and Grouping

VEWs and juice samples were obtained from local supermarkets. The samples were homogenized and three 10 mL aliquots of each sample were collected for the analysis. Samples that were visibly cloudy, contained pulp or sediment were treated with HPLC Grade dimethyl sulfoxide (Sigma); 5 mL of sample was added to 5 mL of DMSO. The samples that were treated with DMSO were then filtered through a 0.45 µm syringe filter; the resulting solution was used in the analysis. 

The samples were grouped as follows: Three name brand VEWs were considered for the investigation, one of the VEW groups also included two low-calorie beverages. Three individual brands were given their own group, these groups were A–C and each number characterizes a different beverage of that brand. Group D included fruit and berry juices. All beverages were purchased in June of 2008. The VEWs that were used are as follows: SoBe Life Water Brand: Shield Life, Calm Life, Challenge Life, Energize Life; Glaceau vitaminwater: Power C, Focus, Balance, Vital-t, Defense, XXX, Low-cal XXX, Formula 50, Charge, Revive, Energy, Low-cal Energy, Essential, Low-cal Essential, Multi-V, Sync; Wegman’s Brand Aqua-V: Fill’er Up, Power-Up, Anti-Up, Put’em Up, Liven Up. Please note that the preceding list does not reflect the manner in which the samples were coded in any way. 

### 2.2. Folin-Ciocalteu Assay

Polyphenol concentrations were measured in triplicate for each sample at 750 nm using the Folin-Ciocalteu reagent (Sigma) diluted 5-fold before use with HPLC Grade water. All polyphenol concentrations were measured in triplicate except for A12–A16, which were measured in duplicate. Catechin was used as a standard and the absorbance measured at 750 nm; all samples were allowed to react with the phenol reagent for 20 min before the absorbance was read. A Thermo Spectronic Genesys 20 spectrophotometer was used for the assay. Results are reported as μM equivalents of catechin. 

### 2.3. Statistical Analysis

All statistical data was gathered using a Mann-Whitney rank sum test for non-normal distributed data; differences were considered statistically significant if *p* < 0.05.

## 3. Results and Discussion

In order to effectively compare the antioxidant capabilities of fruit juices and VEWs, free polyphenols were calculated in μM catechin equivalents. Ascorbate contribution to antioxidant capacity was also removed from the data. A ratio was obtained by using the slope of the standard curve for ascorbate reacting with Folin reagent. This ratio was used to determine the theoretical contribution to the polyphenol absorbance based on the label values of ascorbate. 

**Figure 1:**
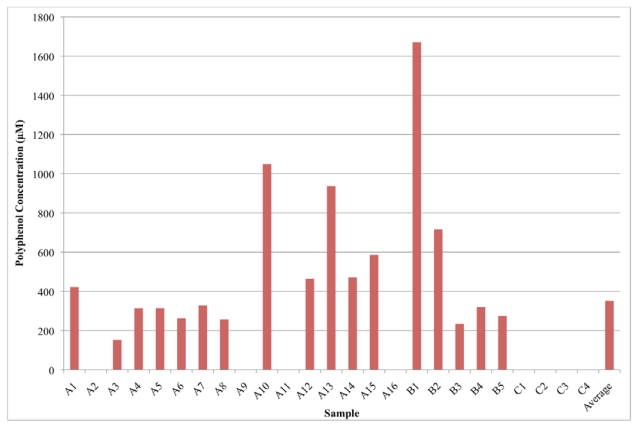
Distribution of free polyphenols in vitamin enhanced waters. The average includes groups A–C. Note A2, A9 A11, A16 and C1–C4 all have 0 μM.

**Figure 2:**
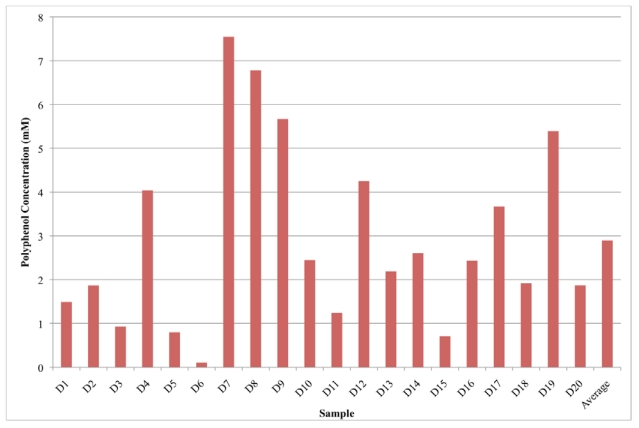
Distribution of free polyphenols in fruit and berry juices.

The results of the analyses are displayed is Figure 1 and Figure 2. Groups A, B, and C designate the different brands of VEWs (Figure 1). Group D represents the fruit and berry juices (Figure 2). The VEWs had varying concentrations, ranging from 1670 μM (B1) to 0 μM, with an average value of 350 μM. The low-calorie VEWs, which contain crystalline fructose, Stevia extract and erythriol as sweeteners, were found to have a wide range of free polyphenols; one beverage contained 0 μM and the other 586 μM, which was above the average for the VEW group inclusive. VEWs could contain polyphenols due to addition of fruit juices and natural flavors as mentioned on the label. Some juices had relatively high concentrations of free polyphenols, such as the cherry blend (D7, 7.54 mM) and blueberry juice (D8, 6.78 mM). Other fruit and berry juices contained relatively smaller concentrations of free polyphenols such as apple juice (D6, 0.110 mM) and grapefruit juice (D15, 0.710 mM). The fruit and berry juices averaged a free polyphenol concentration of 2.90 mM. Overall, out of 20 fruit juices and 25 VEWs, the fruit juices contained, on average, more than eight-times the amount of free polyphenols. The precision of the method is 7.2%, determined by the average standard deviation among all of the samples on which the assay was used to determine free polyphenol concentrations.

One concern of consuming fruit juice as a source of antioxidants is the amount of sugar in the beverage. Bazanno found that consumption of fruit juices may be linked with an increased risk for type 2 diabetes in women [12]. Therefore, the beverages were ranked according to the free polyphenols per serving divided by the total Calories per serving. The rankings are listed in Table 1. Ideally, a beverage should have high polyphenol content with minimal amounts of sugar added in order to be considered a “healthy” beverage. As a result, the total Calories were taken into consideration when ranking the drinks. The samples that occupy the four highest ranks are D17, D8, A15 and D7 (respectively) for free polyphenols per Calorie per serving, suggesting that these sources of antioxidants, although not the highest free number, might be the most healthy to consume, when only considering antioxidants. It should also be noted that most of the beverages that are the most “healthy” with respect to antioxidants per Calorie also have no added sugars or are made to be low in Calories, by either using no supplementary sugar in addition to what is naturally found in the beverage or using artificial sweeteners such as sugar alcohols.

**Table 1:** Free polyphenol content and free polyphenols per Calorie of vitamin enhanced waters (VEWs) and fruit juices based on serving sizes.

Sample Code	Polyphenols per Serving (mg/Serving)	Percent DV per Serving of Vitamin C	Serving Size (mL)	Calories per Serving Size (Cal/Serving)	Free Polyphenols per Calorie (mg/Cal)	Rank ^A^
**VEWs**						
A1	29.4	20%	240.0	50.0	0.59	22
A2	0.00	100%	240.0	50.0	0.00	36
A3	10.6	40%	240.0	50.0	0.21	34
A4	21.8	40%	240.0	50.0	0.44	27
A5	21.8	40%	240.0	50.0	0.44	27
A6	18.3	60%	240.0	50.0	0.37	31
A7	22.8	40%	240.0	50.0	0.46	24
A8	17.8	60%	240.0	50.0	0.36	32
A9	0.00	100%	240.0	50.0	0.00	36
A10	73.1	40%	240.0	50.0	1.46	12
A11	0.00	100%	240.0	50.0	0.00	36
A12	32.3	60%	240.0	50.0	0.65	21
A13	65.2	40%	240.0	50.0	1.30	13
A14	32.8	40%	240.0	50.0	0.66	20
A15 (Low-Cal)	40.8	100%	240.0	10.0	4.08	3
A16 (Low-Cal)	0.00	100%	240.0	10.0	0.00	36
B1	116	100%	240.0	50.0	2.33	8
B2	49.8	100%	240.0	50.0	1.00	16
B3	16.2	60%	240.0	50.0	0.32	33
B4	22.3	40%	240.0	50.0	0.44	26
B5	19.1	40%	240.0	50.0	0.38	30
C1	0.00	100%	240.0	40.0	0.00	36
C2	0.00	100%	240.0	40.0	0.00	36
C3	0.00	100%	240.0	40.0	0.00	36
C4	0.00	100%	240.0	40.0	0.00	36
**Fruit and Berry Juices**						
D1 (15% Cranberry/Grape Juice)	104	100%	240.0	120.0	0.86	17
D2 (27% Cranberry Juice)	130	100%	240.0	120.0	1.08	15
D3 (100% Apricot)	64.8	100%	240.0	130.0	0.50	23
D4 (100% Mango)	281	100%	240.0	130.0	2.16	9
D5 (100% Cranberry)	55.5	100%	240.0	140.0	0.40	29
D6 ^B^ (100% Apple)	7.61	100%	240.0	120.0	0.06	35
D7 (Cherry Blend) ^C^	526	0%	240.0	130.0	4.04	4
D8 ^B^ (100% Blueberry)	472	0%	240.0	100.0	4.72	2
D9 ^B ^(100% Pomegranate)	296	15%	180.0	110.0	2.69	6
D10 ^B^ (100% Pineapple)	126	100%	177.0	100.0	1.26	14
D11 (100% Cherry)	86.7	120%	240.0	120.0	0.72	19
D12 ^B^ (100% Red Grape)	296	100%	240.0	170.0	1.74	10
D13 ^B^ (100% Lime)	3.18	*	5.0	0.0	*	*
D14 ^B^ (100% Lemon)	3.78	*	5.0	0.0	*	*
D15 (30% Grapefruit)	49.4	100%	240.0	110.0	0.45	25
D16 ^B^ (100% White Grape)	170	120%	240.0	70.0	2.42	7
D17 ^B^ (100% Noni)	62.9	*	59.0	8.0	7.86	1
D18 ^B^ (100% Mangosteen)	16.7	*	30.0	10.0	1.67	11
D19 ^B^ (100% Acai)	47.0	*	30.0	15.0	3.13	5
D20 ^B^ (100% Goji)	16.3	*	30.0	20.0	0.81	18

^A^ Ranks were determined by total polyphenols per Calorie based on a single serving; ^B^ Designates beverages with no added sugars; ^C^ Actual content is propriety; * Designates not listed or undefined value.

The statistical analysis of the data was conducted using a Mann-Whitney rank sum test for non–normal distributed data. Since group C contained no polyphenols, based on the analysis, it was excluded from the statistical tests in order to compare beverages that do have free polyphenols. The results of the analysis are reported in Table 2. There was no statistical different between groups A and B for free polyphenol concentration and free polyphenols per Calorie. Comparison of the VEWs to the fruit and berry juices yielded two statistically significant results. The tests found a statistical difference between the groups with respect to both free polyphenol concentration and free polyphenols per Calorie. 

**Table 2:** Summary of statistical data.

Groups Compared	Variable Tested	Resulting *p* Value
A and B	Free Polyphenol Concentration	0.362
A and B	Free Polyphenols/Calorie	0.456
VEWs * and D	Free Polyphenol Concentration	<0.001
VEWs * and D	Free Polyphenols/Calorie	<0.002

* The VEW group includes only groups A and B.

## 4. Conclusions

Although many fruit juices contain high free concentrations of polyphenols, Calories and serving sizes must be taken into consideration when analyzing antioxidant rich beverages. When free polyphenol concentration was analyzed with respect to Caloric content based on a serving size, the beverages D17, D8, A15, and D7 ranked superior. While other fruit juices, on average, contained more than eight-times the concentration of free polyphenols than VEWs, the polyphenol/Calorie ratios were comparable, in most instances. There is a large statistical difference between the VEWs compared to the fruit and berry juices with respect to free polyphenol concentration (*p* < 0.001). The free polyphenols per Calorie data was also statistically different between the VEWs and fruit and berry juices. The fruit and berry juices contained more than twice the amount of free polyphenols per Calorie.
